# Quantification and Evolution of Online Public Opinion Heat Considering Interactive Behavior and Emotional Conflict

**DOI:** 10.3390/e27070701

**Published:** 2025-06-29

**Authors:** Zhengyi Sun, Deyao Wang, Zhaohui Li

**Affiliations:** 1 Graduate School of Information, Production and Systems, Waseda University, Kitakyushu 8080135, Japan; luckysun1999@fuji.waseda.jp; 2 Dept. Library and Information Studies, Fudan University, Shanghai 200433, China; dywang23@m.fudan.edu.cn; 3 School of Maritime Economics and Management, Dalian Maritime University, Dalian 116026, China

**Keywords:** public opinion evolution, public opinion heat, interactive behavior, emotional conflict

## Abstract

With the rapid development of the Internet, the speed and scope of sudden public events disseminating in cyberspace have grown significantly. Current methods of quantifying public opinion heat often neglect emotion-driven factors and user interaction behaviors, making it difficult to accurately capture fluctuations during dissemination. To address these issues, first, this study addressed the complexity of interaction behaviors by introducing an approach that employs the information gain ratio as a weighting indicator to measure the “interaction heat” contributed by different interaction attributes during event evolution. Second, this study built on SnowNLP and expanded textual features to conduct in-depth sentiment mining of large-scale opinion texts, defining the variance of netizens’ emotional tendencies as an indicator of emotional fluctuations, thereby capturing “emotional heat”. We then integrated interactive behavior and emotional conflict assessment to achieve comprehensive heat index to quantification and dynamic evolution analysis of online public opinion heat. Subsequently, we used Hodrick–Prescott filter to separate long-term trends and short-term fluctuations, extract six key quantitative features (number of peaks, time of first peak, maximum amplitude, decay time, peak emotional conflict, and overall duration), and applied K-means clustering algorithm (K-means) to classify events into three propagation patterns, which are extreme burst, normal burst, and long-tail. Finally, this study conducted ablation experiments on critical external intervention nodes to quantify the distinct contribution of each intervention to the propagation trend by observing changes in the model’s goodness-of-fit ($R^{2}$) after removing different interventions. Through an empirical analysis of six representative public opinion events from 2024, this study verified the effectiveness of the proposed framework and uncovered critical characteristics of opinion dissemination, including explosiveness versus persistence, multi-round dissemination with recurring emotional fluctuations, and the interplay of multiple driving factors.

## 1. Introduction

With the rapid development of computer and mobile communication technologies, social networking platforms such as Weibo and WeChat have attracted a large user base, becoming indispensable tools for information dissemination. Social networks have not only become important channels for the public to obtain information and express opinions on hot social issues, but have also evolved into key venues for the generation and fermentation of online public opinion, profoundly transforming the online public opinion ecology. However, due to insufficient technological regulation, it is often challenging to effectively control online public opinion. This shortfall can compromise the scientific handling of emergencies and easily lead to the formation of new waves of opinion that spread further, affecting social harmony and stability [1,2,3]. In some cases, the consequences of these online public opinions can be even more destructive than the events themselves, especially by triggering fermentation and loss of control of public sentiment [4]. Therefore, effectively addressing online public opinion issues is of great significance for creating a secure and reliable online environment. From a practical point of view, the improved monitoring and management of such opinion trends can assist government agencies in crisis intervention, guide enterprises in brand reputation management, and help media outlets optimize their reporting strategies to mitigate misinformation and panic.

To curb or even prevent the spread of online public opinion, it is crucial to understand the mechanisms of opinion dissemination on social networks and to quantify the trends of evolution of opinions. Public opinion heat is a key factor in measuring the degree of attention that an event receives online [5]. It is commonly quantified by counting the number of comments and viewpoints generated by users on social networks. Existing methods for measuring public opinion intensity often overlook the emotional dimension contained within these comments, particularly in the Weibo-based public discourse environment, and, thus, fail to effectively quantify emotion-driven intensity. In addition, users of information dissemination on social networks show high levels of initiative, autonomy, and creativity. Interactions among users and between users and information exhibit multidirectional, multilayered, and high-frequency characteristics. In addition, virtual social networks often correspond to or extend real-world social structures, increasing the uncertainty, randomness, and dynamism of online public opinion. Such a complexity in the dissemination, formation, and evolution of information further hinders research into the laws of public opinion heat evolution and trend prediction.

In response to highly interactive behavior and the uncertainty of online public opinion, this paper proposes an innovative method for quantifying public opinion intensity based on interactive behavior and emotional conflict. By quantifying users’ complex group-interactive behaviors during various stages of the dissemination of online public opinion, this method systematically analyzes the dynamic evolution of topics and emotions. Specifically, an information entropy algorithm is introduced to measure the interaction intensity of public opinion, and the concept of emotional conflict is incorporated into the quantification framework to derive the emotional intensity of public opinion from Weibo. A multidimensional analysis framework that integrates Weibo’s interactive behaviors is further proposed, and the evolutionary characteristics of public opinion intensity during the dissemination process are examined. This approach provides a novel perspective for quantifying emotion-driven public opinion and lays the foundation for a deeper understanding of the mechanisms underlying the evolution of online public opinion. It also offers practical value for stakeholders who need to identify emerging hotspots and manage rapid public sentiment fermentation in a timely manner.

## 2. Literature Reviews

The measurement of public opinion heat is a crucial aspect of information dissemination analysis, designed to represent the potential level of attention and the scope of dissemination of information related to specific issues [6]. Online public opinion often leads to panic, misinformation, and stigmatization of certain groups [7]. Therefore, accurately measuring the heat of online public opinion not only helps in the early detection of potential social risks, but also provides a scientific basis for relevant authorities to better guide public opinion and prevent the spread of social instability. In recent years, as research on social media networks has deepened, this field has experienced significant development, with scholars primarily focusing on the evolution of online public opinion, users’ interactive behaviors, and the measurement of public opinion heat.

### 2.1. Online Public Opinion Dissemination

The dissemination of public opinion on social networks is a crucial avenue for understanding the dynamics of events and predicting real-world behavioral impacts. Research in this area focuses primarily on the evolution processes and factors that influence the dissemination of online public opinion.

From an evolutionary perspective, the dissemination of public opinion is understood as a dynamic lifecycle process typically divided into several stages, from initiation to dissipation. A temporal segmentation approach classifies the process into latency, acceleration, and dissipation stages to analyze the characteristics of information interaction during dissemination [8]. Another method further refines this into four stages: initiation, propagation, maturity, and recession, with each stage requiring different monitoring, early warning, and governance strategies for negative public opinion [9]. From the perspective of agents, online public opinion often forms nodes that aggregate, diverge, and diffuse through interactions between agents, exhibiting three cyclical stages: initiation, diffusion, and recession [10]. For example, in the lifecycle analysis of financial public opinion, the themes of public opinion change in different stages of dissemination, showing significant thematic differences [11]. Furthermore, statistical methods based on the phase-specific word frequency distribution have been used to construct topic models, revealing different evolutionary trends and characteristics of hot topics during the formation and diffusion stages [12]. In general, scholars commonly adhere to the information dissemination lifecycle framework to segment the evolution of public opinion into multiple stages and explore the dynamic characteristics and unique evolutionary patterns of each stage.

From the perspective of influencing factors, the evolution of online public opinion is viewed as the interaction of multiple themes, including topic dynamics, emotional contagion, and interaction. Macrostructural analyses of networks have identified influential individuals, enterprises, and institutions as key nodes in social networks [13]. Further research has highlighted the social value of user-generated word of mouth, finding that influential sources play a significant role in the dissemination of information regardless of their position in the network [14]. For example, studies on Twitter users’ interaction networks have developed models for predicting news heat, showing a significant correlation between the interaction frequency of sharers and information publishers [15]. Research on public opinion hotspots has shown that public opinion heat is closely related to the public nature and interest of the content, and events that involve public interest attract greater attention [16]. In addition, content similarity and attribute modeling of posts have been used to predict the volume of reposts, offering insight into how specific content affects the dissemination outcomes [17].

Despite extensive studies, there remain significant gaps in the research. First, there is a lack of comprehensive insight into the intrinsic behavioral patterns of individuals derived from the attributes of the target user. Second, insufficient attention has been given to the density of node connections and the time lags in interactions during network structural analyses. Third, limited exploration has been conducted on user characteristics and content preferences related to textual content, particularly on platforms like Weibo. Lastly, existing studies fail to adequately address the dynamic changes in user influence over time.

### 2.2. Interactive Behaviors in Online Public Opinion

The rapidity and breadth of the dissemination of social network information highlight the critical role of user interactions in the spread of public opinion. Understanding the influence of user nodes and their roles in dissemination processes is essential to effectively quantify public opinion heat and establish management mechanisms. Current research mainly examines user interactions, node attributes, and their impact on public opinion dissemination, with the aim of uncovering the formation rules and evaluation methods of influence in social networks.

Studies of user attributes often use metrics such as the number of followers, posts, reposts, and mentions to evaluate influence. Basic user information serves as a direct measure of influence [18], while metrics such as follower count, reposts, and mentions are used to quantify the contribution of followers to a user’s influence through weighted calculations [19]. However, studies have shown that the follower count does not necessarily correlate positively with the actual volume of interaction [20]. Thus, solely relying on follower count to measure influence may introduce bias, as these methods consider only intrinsic user attributes while neglecting external contexts.

To improve the precision of the evaluation, recent studies have incorporated interaction attributes within user relationships to construct heat evaluation systems using weighted attributes [21] and have identified highly influential users in the topological structure of information dissemination networks [22]. Quantitative analysis that combines user behaviors such as reposts, comments, and mentions has also been used to measure public opinion heat and influence on dissemination [23]. However, the lack of consideration for the structural characteristics of the community limits a comprehensive understanding of interaction effects.

The topic-specific characteristics of Weibo opinion dissemination have been analyzed by incorporating topic content into the evaluation framework. The influence of the user is calculated using topic similarity and network structures to enhance precision [24,25]. However, these studies neglected user interaction behaviors and related factors. In addition, topic models that integrate post content, reposts, comments, and mentions have been used to identify highly influential users in public opinion dissemination [26], but these models fail to adequately account for the correlation between user interests and topics. Topic model-based algorithms for detecting user interests and ranking users according to dissemination capacity have been deeply explored [27]. Although these studies focus on user interactions, they do not effectively identify users with sustained influence on online public opinion.

In summary, current studies reveal several limitations: first, research based on target user attributes fails to fully uncover intrinsic behavioral patterns; second, analyses of social network structure inadequately consider connection density and temporal differences in interactions; third, the exploration of user characteristics and content preferences related to textual content remains limited. Additionally, insufficient attention has been paid to dynamic changes in user influence over time, highlighting directions for further optimization in online public opinion dissemination research.

### 2.3. Quantification of Public Opinion Heat

Public opinion heat on Weibo refers to the level of attention triggered by specific companies or social events on social media, intertwined with public sentiments, attitudes, and opinions, forming public opinion conflicts. The analysis of the heat of Weibo public opinion helps enterprises identify trends, detect early warning signals, and adjust strategies in a timely manner.

Currently, public opinion heat on social networks is quantified through user behavior metrics such as reposts on Twitter, views on YouTube, and comments on news articles [28]. Specifically, repost volume on Weibo is often used to measure event heat, while articles read on WeChat reflect content heat, and video views measure video heat on platforms like YouTube. Despite their differences in form, these metrics essentially describe information heat. Studies have shown a linear relationship between early and long-term information heat. Early models, such as the SH prediction model based on user access volume to platforms such as YouTube and Digg, accurately predict the long-term heat of online content [29]. Subsequent research revealed that video heat distributions on social networking platforms exhibit power-law characteristics, indicating uneven information heat distribution [30]. The models have since been refined, dividing YouTube video dissemination into multiple time segments to predict future trends based on early heat levels, and multivariate linear regression models improve prediction accuracy [31]. Moreover, the emotional responses of netizens during dissemination processes play a critical role in its profound impact. Some scholars have focused on emotion mining in social networks to understand and guide user emotions during public opinion evolution. For instance, a sentiment analysis of bullet comments on Bilibili highlighted trends and characteristics in user emotions, providing empirical support for emotional dissemination research in social networks [32].

In conclusion, although current heat measurement methods are intuitive and practical, they lack sufficient consideration of emotional dimensions in comments, failing to quantify emotion-driven heat changes effectively. Furthermore, heat evolution is influenced by multiple factors, such as content and user interactions, and exhibits diverse dissemination patterns. Future research should comprehensively analyze the evolution of public opinion heat by combining interactive behaviors and emotional dynamics.

### 2.4. Social Media Data Analysis Based on Clustering Algorithms

In recent years, clustering algorithms have been applied in the field of social media data analysis. Adnan et al. [33] used the Word2Vec model to transform tweet texts into 100-dimensional word vector representations and applied principal component analysis (PCA) to reduce the high-dimensional data to a two-dimensional space. They then used the K-means to cluster the data, thereby identifying different groups of social media users [33]. Alsayat et al. [34] proposed an optimized K-means framework to improve the accuracy of user group identification. Specifically, they first utilized a genetic algorithm to optimize the initial centroids of the K-means and then introduced an optimized cluster distance (OCD) method to further increase inter-cluster distances while reducing intra-cluster variance. In addition, they conducted an in-depth segmentation of social media user groups based on authority score, hub score, and sentiment orientation [34]. Zul et al. [35] introduced a sentiment analysis framework that combines K-means with the Naïve Bayes classifier for sentiment classification. The study found that, compared to the combined K-means and Naïve Bayes approach, using Naïve Bayes alone yielded better performance in sentiment classification tasks [35].

To address the high spatial complexity and randomness of initial centroids in the traditional K-means, Madhuri et al. [36] proposed an optimized fuzzy means clustering algorithm. This method randomly initializes cluster centers and iteratively updates them based on the degree of word matching and associated weights with data points until convergence. The experimental results showed that this optimized algorithm improved the accuracy of the clustering while effectively reducing time and computational complexity [36]. Qi et al. [37] proposed a model that integrates K-means with particle swarm optimization (PSO) to optimize public opinion dissemination analysis on social media. They used PSO to optimize the initial centroids of the K-means, overcoming the tendency of traditional K-means to become trapped in local optima. The experimental results demonstrated significant advantages in clustering purity [37].

The aforementioned studies highlight the diverse applications and continued refinement of clustering algorithms in social media analysis. However, most existing approaches predominantly emphasize static attributes such as keywords and sentiment labels or singular aspects of dissemination dynamics, thereby neglecting the intricate interplay between user interactions and emotional factors during opinion propagation. Furthermore, these methods fail to capture nuanced fluctuations jointly driven by interactive behaviors and emotional variations, limiting their ability to elucidate the deeper dynamics and inherent mechanisms of the evolution of public opinion. Thus, an integrated approach considering both interactive behaviors and emotional conflicts is required for a comprehensive understanding of the complex patterns underlying the dissemination of public opinion.

## 3. Construction of Public Opinion Heat Index Considering Interactive Behavior and Emotional Conflict

**Figure 1:**
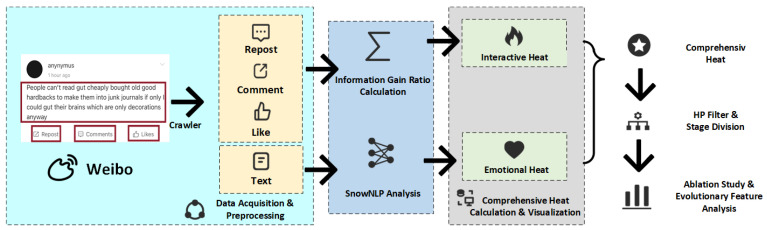
The whole process of the proposed method.

In this section, we quantify the number of interactive behaviors for posts under a topic using the information gain ratio to define the interaction intensity at the current stage of public opinion and propose an interaction intensity index for public opinion. Our proposed framework begins with the collection and preprocessing of Weibo data, including posts, reposts, comments, and likes. Next, interaction heat is quantified by applying the information gain ratio to weight different interaction attributes, while emotional conflict is derived via SnowNLP. These two metrics are then integrated into a comprehensive heat index, which is subsequently decomposed using the Hodrick–Prescott filter (HP filter) to distinguish trend and cyclical components. Then, we extract six key quantitative features from the filtered series, the number of peaks, the time of the first peak, the maximum amplitude, the duration of the decay, the peak emotional conflict, and the overall duration, and we apply K-means to classify events into different dissemination patterns. Finally, we conduct ablation experiments on critical external intervention nodes to quantify the distinct contribution of each intervention to the propagation trend and format additional classification or management strategies. The entire process is illustrated in Figure 1.

### 3.1. Construction of Public Opinion Heat Index Model Based on Interactive Behavior

Public opinion heat reflects the intensity of user engagement in event discussions and dissemination, as well as the scope of the influence of the event on social networks. On platforms like Weibo, user interactive behavior relies on specific posts, and the impact of individuals and content within a public opinion topic ultimately manifests in the quality of post interactions. In major sudden or controversial events, the corresponding posts typically see higher numbers of comments, reposts, and likes, resulting in increased public opinion heat generated by user interactions [38].

#### 3.1.1. Modeling of the Public Opinion Interaction Heat Index Based on Information Gain Ratio

Public opinion heat can be defined by the number of reposts, comments, and likes on posts within a unit of time. It represents an incremental metric where each type of interactive behavior positively contributes to the overall trend of public opinion heat. To assign appropriate weights to different interactive behaviors, this study adopts an information-theoretic approach, using the concept of categories to represent sample set distributions. In this approach, entropy measures the degree of disorder in a sample set: minimal entropy occurs when all samples belong to a single category, while a higher entropy indicates a more even distribution across categories. This principle is applied to assess how effectively each feature reduces system uncertainty. Features that partition the data into more homogeneous subsets, thereby reducing entropy, are assigned higher weights. Thus, categories serve as an information-theoretic tool to evaluate the explanatory power of each feature in modeling the intensity of public opinion interaction.

Suppose that an event has an initial outbreak time $t_{0}$. We can observe interactive behaviors during the time interval *t* following the outbreak. We define E(t) as the set of all posts related to this public opinion event within the current time window *t* and e(i,t) as an individual post within that set, as expressed below:

(1) E(t)={ e(i,t)∣i=1,2,3,…,n},0≤t≤T

(2) e(i,t)={{ r(i,t),c(i,t),l(i,t)}∣i=1,2,3,…,n},0≤t≤T

where r(i,t), c(i,t), l(i,t) represent the repost, comment, and like counts of the post e(i) within the time window *t*, and *T* denotes the entire lifecycle of public opinion dissemination.

Since interactive behavior features contribute differently to public opinion heat, they require different weight values. This study uses the information gain ratio to determine these weights. Before delving into the information gain ratio, it is crucial to define both the information entropy and the information gain. Information entropy quantifies the amount of uncertainty in a sample set. A set is considered disordered when it contains multiple categories, while it is pure when all samples belong to one category [39]. Thus, information entropy reflects the degree of disorder or purity in a sample set. The formula is shown below:

(3) $$Ent\left(S\right)=-\sum_{i=1}^{m}p_{i}\log p_{i}$$

where *m* represents the number of category labels in the sample set, *i* denotes a specific category label in the sample set, and $p_{i}$ is the proportion of samples belonging to the *i*-th category. A higher information entropy indicates that the sample set’s classifications are more dispersed and impure, whereas a lower information entropy implies that the classifications are more concentrated and pure. Therefore, information entropy is measured for the entire feature set. After partitioning the sample set based on a given feature, the more changes occur in the category the sample set belongs to, the greater the amount of information carried by that category. Consequently, the formula for the information gain contributed by the feature *A* to the sample set *S* is given by

(4) $$Gain\left(S,A\right)=Ent\left(S\right)-\sum_{v=1}^{V}\frac{\left|S_{v}\right|}{\left|S\right|}\;Ent\left(S_{v}\right)$$

here, Gain(S,A) represents the information gain, *S* denotes the sample set, *A* is a feature of the sample set *S*, *V* indicates that the feature *A* has *V* distinct values with *v* being one such value, and $S_{v}$ is the subset of *S* where the feature *A* takes the value *v*. Thus, $s_{v}$ is a specific element or data point from the subset $S_{v}$. From the formula, the information gain tends to favor features with numerous values. However, if we consider the user ID as a feature, since each ID is unique, the resulting information gain would be extremely high, implying that this feature is exceptionally suitable for splitting the sample set.

However, in practice, using such a feature to partition the dataset is meaningless. To counteract the negative impact of this bias, the concept of information gain ratio is introduced, calculated as follows:

(5) $$Gain\text{-}Ratio\left(S,A\right)=\frac{Gain(S,A)}{I(A=a_{j})}$$

(6) $$I\left(A=a_{j}\right)=-\sum_{i=1}^{k}p_{ij}\;\log p_{ij}$$

where Gain-Ratio(S,A) represents the information gain ratio of the feature *A*, Gain(S,A) denotes the information gain of feature *A*, and $I(A=a_{j})$ indicates the purity of the values of the feature *A*. If *A* has only a few distinct values, then the purity of *A* is relatively high. In contrast, as the number of values for *A* increases, the value of $I(A=a_{j})$ also increases, ultimately resulting in a lower information gain ratio. In Formula (6), $p_{ij}$ represents the probability that the *i*-th class occurs for feature *A* when *A* takes the value $a_{j}$. Specifically, $p_{ij}$ is the proportion of instances in class *i* within the subset of data where $A=a_{j}$. This term is used to compute the entropy of the feature *A* when it takes the value $a_{j}$, quantifying the uncertainty or impurity of that subset.

By calculating the information gain ratio of each feature, we obtain a reference indicator for assessing its influence on interaction heat. The weight allocation method based on the information gain ratio is as follows:

(7) $$w_{i}=\frac{Gain\text{-}Ratio(S,A_{i})}{\frac{1}{n}\sum_{i=1}^{n}Gain\text{-}Ratio\left(S,A_{i}\right)}$$

here, $w_{i}$ denotes the weight of the feature $A_{i}$ in the sample set. A higher weight for the feature $A_{i}$ indicates that it has a greater impact on the classification results. $Gain\text{-}Ratio(S,A_{i})$ represents the information gain ratio of feature $A_{i}$, and $\frac{1}{n}\sum_{i=1}^{n}Gain\text{-}Ratio\left(S,A_{i}\right)$ denotes the average information gain ratio of all features in the sample set.

Within a given time window, the interaction heat carried by post e(i) is defined as follows:

(8) $$HB\left(i,t\right)=w_{r}\times f_{r}\left(i,t\right)+w_{c}\times f_{c}\left(i,t\right)+w_{l}\times f_{l}\left(i,t\right),\;0\leq t\leq T$$

where $f_{r}\left(i,t\right)$, $f_{c}\left(i,t\right)$, and $f_{l}\left(i,t\right)$ represent the proportions of reposts, comments, and likes within the time window *t*, respectively. Specifically, $w_{r}$ reflects the importance of reposts, $w_{c}$ indicates the contribution of comments, and $w_{l}$ represents the role of likes in the calculation of HB(i,t). These weights allow different forms of engagement to be prioritized based on their significance in the analysis.

The overall interaction intensity HB(t) for posts under the same topic is as follows:

(9) $$HB\left(t\right)=\sum_{e\in E}HB\left(e,t\right),\;0\leq t\leq T$$

#### 3.1.2. Definition of the Public Opinion Interaction Heat Index

By quantifying the various types of interaction frequencies for posts under the same topic, the heat generated by user interactive behaviors in public opinion events can be measured. In advertising theory, the brand development index (BDI) tests the dynamic real-time performance of a brand level and illustrates the brand’s development relative to a certain baseline [40]. Drawing inspiration from this concept, we propose a heat index. By setting the heat value of the first time window as the baseline and then tracking the changes in subsequent index values, we can monitor the development trends of public opinion and investigate the evolutionary patterns of hot topics of public opinion. Thus, the heat index $I_{C}$ of public opinion at time window *t* is defined as

(10) $$I_{C}=I_{0}\times \frac{{HB}_{t}}{{HB}_{\mathrm{base}}},$$

here, ${HB}_{t}$ represents the interaction heat of public opinion during the *t*-th time window, ${HB}_{\mathrm{base}}$ denotes the interaction heat at the event’s initial release, and $I_{0}$ is the baseline value of the heat index for the first observed time window of the public opinion event, set as $I_{0}=100$. This allows us to discern the pattern of how public opinion heat varies over time and to conduct corresponding evolutionary analyses.

### 3.2. Construction of Public Opinion Heat Index Considering Emotional Conflict

Netizen sentiment refers to the emotions triggered or generated among internet users (netizens) when they become aware of specific social events. In the new media environment, netizens influence the evolution of such events by posting, reposting, and commenting. Their interactive behaviors on social networks are often driven by their underlying sentiment orientation. As a result, sentimental characteristics exert a strong driving force, effectively stimulating self-initiated and conformity-driven dissemination intentions, which, in turn, influence the spread of public opinion. Given that the Internet is a medium characterized by immediacy, interactivity, and high social connectivity, the contagious nature of emotions, coupled with the diffusion effects of the Internet, leads to emotional homogenization between individuals within a group. The aggregation of these emotions can yield unpredictable consequences [38,41]. Therefore, when examining the process and intensity of public opinion dissemination, it is necessary to consider the impact of sentiment.

This study proposed employing SnowNLP for sentiment classification on a Chinese comment dataset. We utilized Word2vec embeddings to segment each Chinese comment into words and convert them into matrix representations based on their corresponding word embeddings. These matrices were then input into SnowNLP, where convolution and pooling operations extracted sentence-level features. A classifier subsequently distinguished between positive and negative sentiments, allowing us to obtain a set of all sentiment-oriented texts related to public opinion topics over a given period, denoted as G(t).

(11) $$G\left(t\right)=\{\;\left(m_{i},g_{i}\right)|i=1,2,3,\ldots ,n\},\;0\leq t\leq T$$

In this context, $g_{i}$ denotes the sentiment orientation value corresponding to the text $m_{i}$ within the current time window *t*. In the literature review, it is observed that scholars generally focus on the straightforward determination of text-based sentiment tendencies when analyzing the affective features of public opinion. Although such approaches can uncover the user emotions behind interactive behaviors, they often fail to consider the contradictions and blending that arise when different emotional expressions concerning the same topic collide. Furthermore, as netizens become more adept at public relations, and with the rise of fan economies, the presence of “water armies,” “ghost followers,” marketing accounts, and clickbait users on Weibo continues to increase. In the same post, one often finds a large number of identical reposts and comments, which poses a significant challenge for relevant departments seeking to understand the genuine viewpoints and attitudes of netizens about an event.

To address this issue, the concept of “emotional conflict” is introduced to identify the fundamental driving force behind the development and evolution of public opinion. When confronted with the same public opinion event, users may express divergent viewpoints, resulting in emotional conflict. The greater the degree of emotional conflict, the more intense the discussion becomes, thereby accelerating the rapid fermentation and escalation of public opinion. Consequently, based on the analysis of textual sentiment tendencies, this section introduces emotional conflict to better identify the underlying emotions of Weibo users in the online environment.

For discussions centered on the same event, a greater degree of emotional conflict among netizens indicates more intense debate, which, in turn, suggests an increased likelihood that subsequent public opinion will intensify and the situation will escalate, thus further driving an upsurge in public opinion heat. In statistical analysis, variance is commonly used to measure the degree of dispersion within a dataset. A larger variance signifies greater dispersion of data values, reflecting heightened irregularity and complexity. This property, which characterizes differences in data, aligns conceptually with the notion of emotional conflict. Therefore, in this study, the variance of netizen sentiment orientation values regarding public opinion texts is employed to represent their emotional conflict, denoted as HE.

The magnitude of HE reflects the extent of emotional divergence and contradiction that users express toward the same online event. A larger HE value indicates more complex and continuously entangled emotions among netizens, which can trigger further debate and escalate public opinion conflicts. Conversely, a smaller HE value suggests that users’ emotions are more unified, and the diminished contention points contribute to easing the tension in public opinion. In summary, for a given post e(i) pertaining to a certain topic, the intensity of emotional conflict in public opinion that it carries at time step *t* is defined as follows:

(12) $$HE\left(e_{i},t\right)=\mathrm{Var}\left(\sum_{i=1}^{n}c_{i}\right)$$

where $c_{i}$ represents the sentiment intensity or value of the *i*-th user at a given time step. It quantifies the emotional contribution of each user to the overall sentiment, with the variance reflecting the fluctuation and distribution of sentiments within the time window *t*.

Within a given time window *t*, the emotional heat HE(t) carried by all posts under the same topic is defined as follows:

(13) $$HE\left(t\right)=\sum_{e\in E}HE\left(e,t\right)$$

referring to the calculation method illustrated in Equation (10), the emotional heat index $I_{E}$ of the public opinion at time window *t* is obtained as follows:

(14) $$I_{E}=I_{0}\times \frac{HE_{t}}{HE_{\mathrm{base}}}$$

where ${HE}_{t}$ denotes the emotional heat of public opinion during the *t*-th time window, while ${HE}_{\mathrm{base}}$ represents the initial interaction heat of the event at the time of its initial release. Given $I_{0}=100$ as the baseline for the emotional heat index in the first observed time window, we can thereby compute the emotional heat value of the online event. This provides a foundational basis for investigating the propagation patterns of public opinion and the evolution of emotional heat.

### 3.3. Public Opinion Comprehensive Heat Index

Building upon aforementioned foundations, this study primarily examines public opinion propagation from two perspectives: the user interactive behavior and the emotional dimension embedded within these interactions. In the early stages of a public opinion event, netizens, aiming to gain a deeper understanding of the incident, participate in its dissemination by reposting, commenting, and other interactive behaviors. The texts generated through these interactions have a user’s sentiment orientation. As the “heat” of the public opinion ferments, it reinforces netizens’ interactive behavior and emotional conflict.

At a certain stage in the evolution of online public opinion, there may be relatively few participating netizens, yet the level of controversy (i.e., emotional conflict) is high. This situation represents a scenario of low interaction intensity but high emotional heat. Such ideological contradictions and emotional conflicts often serve as a fuse, igniting future intensification of public opinion. Hence, it is crucial to pay attention to the dynamic fluctuations in emotional heat at an early stage. Conversely, there are also situations where a large number of netizens are involved but the emotional conflict is minimal. This reflects a scenario of high interaction intensity but low emotional heat. In this case, although netizens’ sentiment orientations tend to be unified, the intrinsic prominence of the event’s topic maintains a high level of interaction intensity.

In summary, interactive behavior heat and emotional conflict heat jointly produce overall public opinion heat in a complementary manner. Therefore, this study proposes a comprehensive public opinion heat model based on both interactive behavior and emotional conflict, as detailed below:

(15) $$I=\mathrm{minmax}\left(\mathrm{minmax}\left(I_{C}\right)\times I_{E}\right)$$

in the equation, *I* denotes the comprehensive public opinion heat, $I_{C}$ represents the interactive behavior heat, and $I_{E}$ signifies the emotional conflict heat. The term minmax refers to the Min-Max normalization method, which scales data to a fixed range, typically [0,1], ensuring that interactive behavior and emotional conflict features contribute equally to measuring public opinion dissemination heat.

## 4. Empirical Analysis

### 4.1. Dataset Description

The data for this study was sourced from the Weibo platform, aiming to systematically capture the interactive behaviors and emotional responses that occur during the online dissemination of public opinion events. To ensure comprehensive coverage of the entire propagation cycle, data were collected using Python 3.7.0 and requests 2.32.4 in combination with the Weibo API. Public opinion dynamics were recorded at hourly intervals. The collected data included primary post information (publisher ID, publication time, number of comments, number of reposts, number of likes), as well as the contents of comments and reposts, and user sentiment data.

To ensure data quality, all acquired data were cleaned and deduplicated to eliminate irrelevant information and “astroturfing” (water army) content. Following text preprocessing and word segmentation, sentiment analysis was performed using SnowNLP 0.12.3 to generate sentiment intensity indicators within defined time windows. Although SnowNLP provides fundamental coarse-grained sentiment classification, it has limitations in capturing the nuanced, fine-grained dynamics of sentiment evolution over time [42,43]. Therefore, this study further tracked sentiment changes across multiple time windows to analyze sentiment dynamics in greater detail. Additionally, sentiment trends were cross-analyzed with interaction behavior intensity, measured by the volume of comments, retweets, and likes, enhancing the interpretability and explanatory power of the sentiment evolution.

In studying the evolutionary patterns of public opinion, it is essential to select representative events for detailed analysis. By examining the dissemination pathways and topic characteristics of these typical cases, both common and distinct patterns in the propagation and structural features of public opinion can be objectively revealed. Accordingly, this paper selects six high-impact hotspot events from three distinct categories in 2024 as research samples (see Table 1). These six public opinion events serve as core research subjects for conducting an in-depth exploration and quantitative analysis of the evolution patterns and trends in public opinion intensity.

Table 1 shows an overview of the classification, and key indicators reveal three main categories of events. Under Enterprise Survival, E1 (the “‘Dong Bei Yu Jie’ Livestream Sales Failure”) centers on public opinion triggered by failed livestream marketing, typically showing multiple rounds of dissemination and a relatively long lifespan, whereas E2 (“‘Crazy Xiao Yang’ Livestreaming Meicheng Mooncakes”) focuses on promotional activities for e-commerce or festival products, often garnering explosive attention in the short term but declining rapidly. In the Social Livelihood category, S1 (the “315 Sausage Scandal”) represents a classic instance of food safety exposure, in which official media disclosures timed around Consumer Rights Day (15 March) usually generate a strong yet singular burst of public attention, while S2 (the “Oil Tanker Mixing Edible Oil Incident”) involves food safety concerns that may lead to secondary or even multiple rounds of fermentation if further investigations or evidence arises. Lastly, Culture and Sports events include C1 (“Li Ziqi’s Comeback”), wherein a prominent figure’s return can swiftly capture widespread focus and peak discussions within a short period, and C2 (“Wu Liufang Incident”), which skews toward personal controversies or negative topics and may escalate into multi-round conflicts driven by ongoing involvement of fans, media outlets, and other public figures.

### 4.2. Empirical Analysis of the Public Opinion Heat Index Model

#### 4.2.1. Analysis and Visualization of the Public Opinion Interaction Heat Index

Netizen participation in online public opinion typically takes the form of interactive behaviors, such as reposts and comments. These behaviors serve as the most direct driving force propelling the development and spread of public opinion. Weibo posts, as the main carriers of these interactions, provide the most intuitive reflection of an event’s online prominence, as measured by the volume of posts related to a particular topic and the number of reposts and comments they generate.

**Figure 2:**
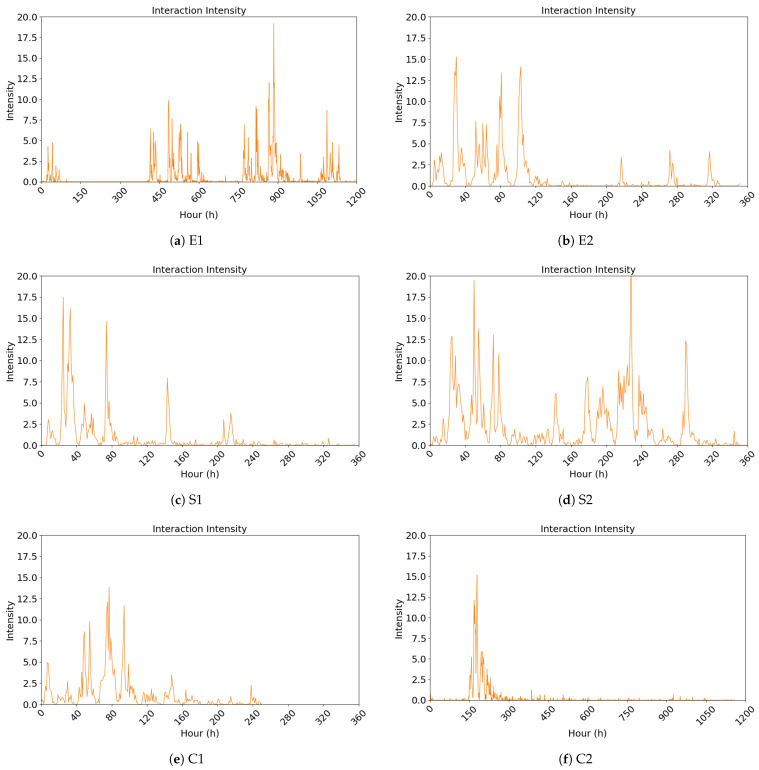
Public opinion heat index based on interactive behaviors.

Based on the interaction-based public opinion heat measurement model introduced in Section 3, a series of public opinion interaction intensity values are computed at hourly intervals. The results of six events are visualized in Figure 2. For comparability and ease of observing differences in intensity and duration across events, the axes scales for time (hours) and intensity are standardized across all subplots. For consistency, events with a duration of 360 h or less are displayed with a time axis capped at 360 h, while those exceeding 360 h are shown with a time axis extending to 1200 h.

From the above figures, although the six events display varying interaction intensity values, they all generally follow a pattern of an initial increase followed by a subsequent decline, with several fluctuations appearing at later time nodes. Moreover, the interaction intensity of these events can reach peak levels within a short period, indicating that these events possess characteristics that rapidly attract and concentrate on public attention.

#### 4.2.2. Analysis and Visualization of the Public Opinion Emotional Conflict Heat Index

**Figure 3:**
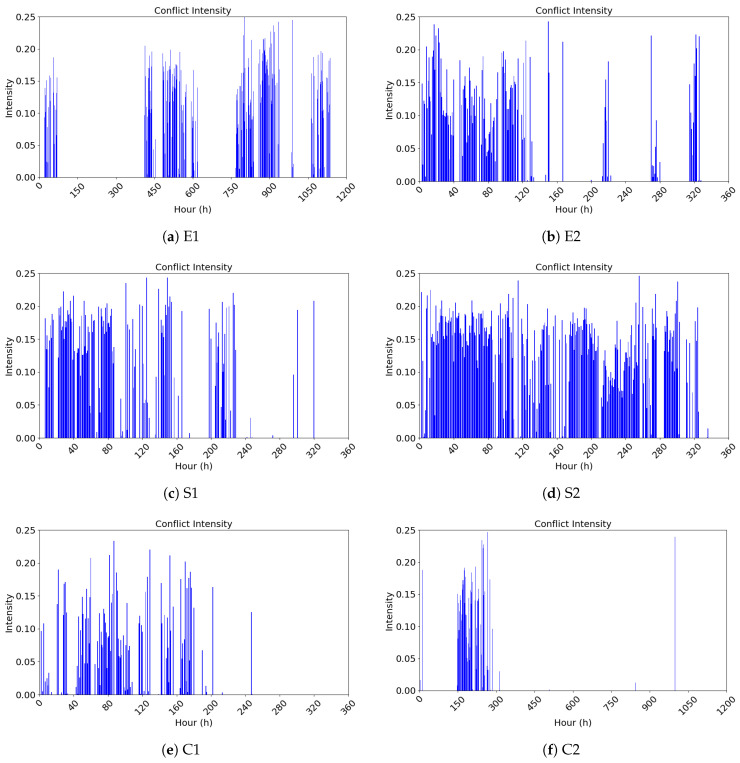
Public opinion heat index based on emotional conflict.

When online media report on a certain public opinion event, it often draws the attention of netizens to that event. As attention grows, it is frequently accompanied by emotional conflict among netizens. The greater the emotional conflict among netizens, the more likely they are to engage in online debates for the dominant narrative. Such “violence against violence” approaches ultimately intensify conflicts, which, in turn, influence the extent and manner in which online media continue to report on the event, potentially leading to a concentrated eruption of public opinion. We introduce the sequence variance to represent the extent of emotional conflict among netizens on a given event. We model the emotional heat for each event, generating an hourly series of emotional heat values based on emotional conflict. The visualization results are shown in Figure 3.

#### 4.2.3. Analysis and Visualization of the Comprehensive Public Opinion Heat Index

**Figure 4:**
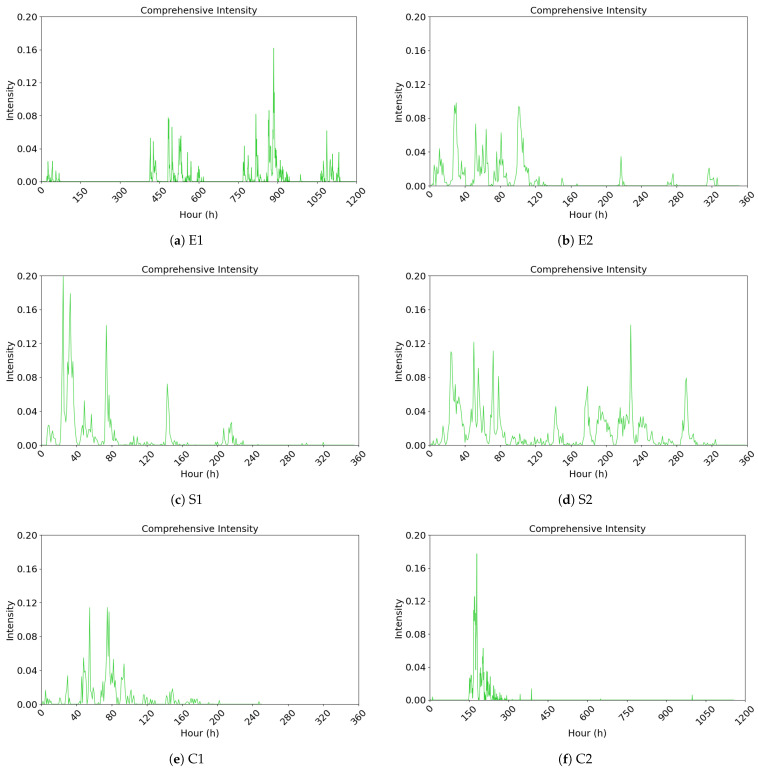
Public opinion heat index based on interactive behaviors and emotional conflict.

Finally, we integrate netizen interactive behavior and emotional conflict to construct a public opinion heat measurement model. Inspired by the concept of information gain ratio, we measure various interactive behaviors to reduce the subjectivity often seen in existing public opinion heat indicators. Meanwhile, we employed SnowNLP to identify user sentiment tendencies and calculate sentiment intensity values. Considering that divergent sentiment orientations can amplify public opinion, we introduce an emotional conflict metric to form a comprehensive public opinion heat index encompassing interactive behaviors and emotional conflict (see Figure 4). This index serves as a foundational model for subsequent research into public opinion evolution patterns.

### 4.3. Dividing Online Public Opinion Evolution Stages Based on HP Filter

**Figure 5:**
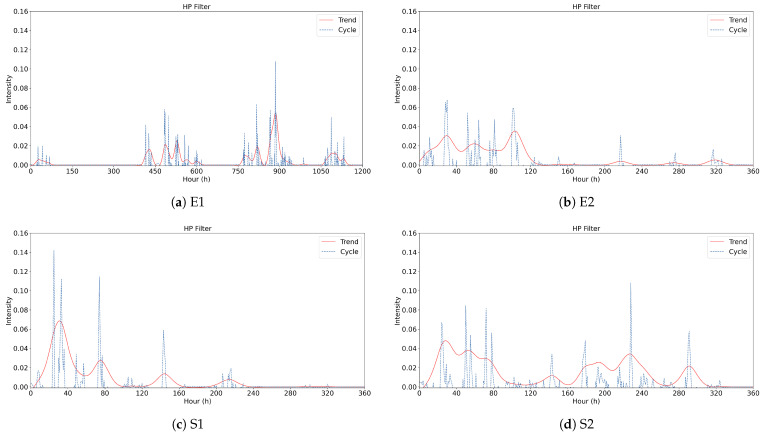

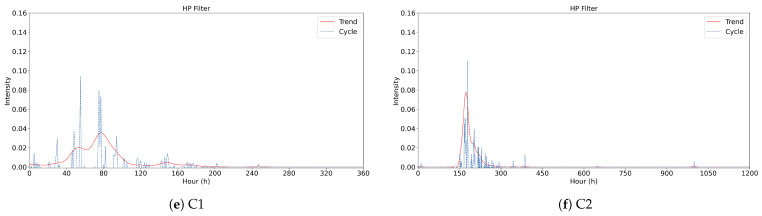
HP filter analysis results.

In this study, in order to more accurately identify the propagation stages of public opinion, we adopt the HP filter method to decompose the time series of public opinion heat in order to extract the long-term propagation trend of the event and distinguish the short-term fluctuations. HP filter is a smoothing technique widely used in time series analysis, which can effectively remove high-frequency fluctuations in data, thus revealing the core trend of public opinion propagation. Under this method, the time series of public opinion $y_{t}$ is decomposed into smoothed trends $g_{t}$ and short-term fluctuations $c_{t}$.

(16) $$y_{t}=g_{t}+c_{t}$$

where $g_{t}$ represents the long-term propagation trend of the event, reflecting the overall evolution of public opinion, while $c_{t}$ reflects the impact of short-term local changes, such as the impact of breaking news, participation of opinion leaders, official announcements, etc., on the heat of public opinion. HP filter calculates the smoothed trend $g_{t}$ by minimizing an objective function, which consists of two parts: the first part minimizes the error between the original data $y_{t}$ and the trend $g_{t}$ to ensure that the trend curve fits the data as closely as possible; the second part imposes a smoothing constraint on the trend change to prevent the trend curve from fluctuating dramatically. The degree of smoothing is controlled by the parameter μ; the larger μ is, the smoother the trend curve is, reflecting the long-term trend; the smaller μ is, the closer the trend curve is to the original data, capturing more short-term fluctuations. This value μ=1600 is selected as the smoothing parameter in this study, which is usually used for quarterly economic data analysis, and is also applicable to high-frequency time-series data of social media opinion dissemination. Finally, the results are shown in Figure 5, where the trend line indicates the long-term trend of public opinion and the cycle line indicates the short-term fluctuation.

### 4.4. Robust K-Means and Stability Validation of Propagation Patterns

#### 4.4.1. K-Means-Based Public Opinion Clustering

In order to objectively analyze public opinion events, based on the results of HP filtering analysis, the six key features in Table 2 were extracted, taking into account the interactive behavior (the intensity of reposts, comments, and likes) and the degree of emotional conflict (the ratio of positive to negative emotions and the frequency of emotional fluctuations); the calculation results are shown in Table 3. These features include different dimensions such as the peak, duration, and decay of an outbreak [44], thus retaining temporal information about the evolution of an event while avoiding the dimensional bias inherent in simple terminological frequency or sentiment scoring methods when comparing different events.

The classification of event propagation patterns is divided mainly into supervised and unsupervised models [45], where supervised learning refers to the use of training samples with labels (known categories) to learn mapping relationships to predict the categories of new samples, while unsupervised learning mines the intrinsic structure on unlabeled data, and it is commonly used to automatically classify public opinion events according to their similarity using methods such as clustering and topic modeling. However, public opinion research often lacks a unified and comprehensive manual annotation framework, which makes it difficult to directly adopt supervised models [46]. On the one hand, different domains (e.g., corporate crises, public security incidents, cultural and entertainment topics) apply inconsistent criteria for “risk levels”. On the other hand, the subjectivity and high cost of manual annotation limit the construction of large-scale labeled corpora. In contrast, unsupervised models such as K-means and Ward’s hierarchical clustering are regarded as standard baseline methods for clustering analysis due to their fully unsupervised nature, computational stability, and interpretability of results [47,48,49], and they have been widely applied in data mining domains including consumer research, bioinformatics, and public opinion classification [50]. Accordingly, this study employed K-means to cluster six public opinion events, determined the optimal number of clusters (*k*) using the elbow method and silhouette coefficient, designed noise-perturbation experiments to assess model robustness, and validated the clustering results with Ward’s hierarchical clustering.

The six features listed in Table 2 were used as input vectors and subjected to Z-score normalization to eliminate units and render the data dimensionless; Z-score normalization (also known as zero-mean normalization) is one of the most common techniques for transforming variables into a dimensionless form [51]. Each raw feature $x_{i}$ is first centered by subtracting its sample mean μ and then scaled by its standard deviation σ, yielding the normalized score $z_{i}$:

(17) $$z_{i}=\frac{x_{i}-\mu }{\sigma }$$

After transformation, each feature distribution has a mean of 0 and variance of 1, completely eliminating units and rendering the data dimensionless while preserving the original relative differences (i.e., deviations of each observation from the mean). This prevents features with larger magnitudes from dominating distance calculations or gradient updates and, thus, avoids clustering bias.

**Figure 6:**
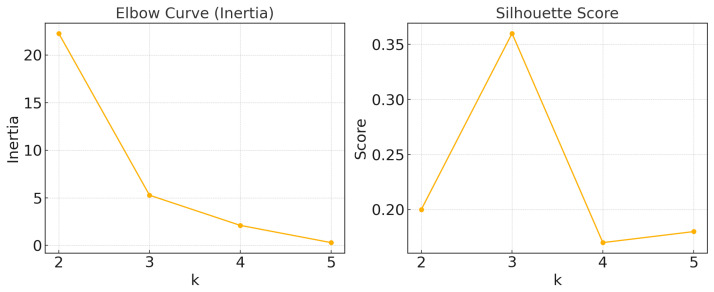
Elbow curve and sihouette score of K-means.

To determine the optimal number of clusters *k*, two complementary evaluation metrics, the elbow method and the silhouette coefficient, were employed. The elbow method measures the compactness of the clusters by calculating the inertia (sum of squares) of the clusters, with lower inertia values indicating more compact clusters. The silhouette coefficient evaluates cluster separation by comparing the average distance between each data point and other points within its cluster to the average distance between the point and points in the nearest neighboring cluster. A higher silhouette coefficient indicates better-defined clusters with a clearer separation. K-means was performed on the normalized dataset (with all features standardized to mean 0 and variance 1) for *k* ranging from 2 to 5. The results are presented in Figure 6 and Table 4.

As shown by the elbow curve in Figure 6, there is a clear “elbow” at *k* = 3, indicating that when the number of clusters exceeds three, the improvement in cluster compactness resulting from additional increases in *k* gradually decreases. Meanwhile, the contour coefficient reaches a maximum value of 0.36 at *k* = 3, which indicates that the clustering structure has struck a good balance between intra-cluster consistency and inter-cluster separation. Although the value of contour coefficient has not reached a high level, which indicates that the separation of the cluster structure is acceptable but not particularly strong [52], the choice of *k* = 3 as the optimal number of clusters for the subsequent analysis is still supported. Furthermore, to further validate the reasonableness of this choice, additional validation in conjunction with a hierarchical clustering approach based on Ward links is recommended [53,54].

To further corroborate the number of clusters (*k*), we independently applied Ward’s minimum-variance hierarchical clustering to the same standardized features. First, the trends of the average silhouette coefficient for Ward across *k* peaked at 0.39 for *k* = 2, remaining high at 0.36 for *k* = 3, and dropping rapidly to approximately 0.18 for *k* ≥ 4, indicating that overpartitioning substantially degrades cluster cohesion and separation. Next, we computed the Calinski–Harabasz (CH) index (Table 4). When *k* increased from 2 to 3, the CH value rose sharply from 4.10 to 8.74, showing that three clusters significantly improved between-cluster dispersion while maintaining within-cluster compactness. Although CH increased slightly to 10.74 for *k* = 4 and *k* = 5, the corresponding average silhouette coefficient had already fallen to 0.18, signaling the emergence of “fragmented clusters” whereby gains were driven by splitting very small samples rather than true structures. Considering both the CH index and silhouette coefficient, the three-cluster solution balanced internal validity and avoided oversegmentation, making it the optimal Ward partition. Finally, we used ARI to measure label concordance between the two methods. At *k* = 3, ARI reached 1.00, indicating stability by both algorithms. Integrating internal validity metrics and practical interpretability, we, thus, confirmed the reliability of the three-cluster K-means solution as reflecting the robust structure revealed by the data. Consequently, *k* = 3 was selected as the optimal cluster count. The resulting clusters are shown in Table 5: cluster A corresponds to S2; cluster B comprises E2, S1, C1, and C2; cluster C corresponds to E1.

In recent years, scholars have conducted extensive research on the dynamics of information propagation in social media. On platforms such as Facebook and YouTube, viral posts exhibit a single sharp peak followed by rapid decay—a “sudden-type” curve characterized by low residual engagement and only one global maximum [55,56], thereby forming an “extreme burst” pattern. Although many public opinion events reach only limited peak intensity, they can be reactivated multiple times after engagement falls to zero due to external triggers, resulting in a power-law long tail that necessitates ongoing risk management [57]; this “long-tail burst” pattern, thus, emerges as an independent category.

Moreover, empirical evidence from q-exponential decay studies on Chinese BBS data and the burst-decay model applied to Twitter data from the Italian elections both indicate that trajectories with moderate peaks and repeated fluctuations within a concentrated time frame are most common; during the burst period, the trend line shows multiple local maxima with no long-tail behavior [58,59], constituting a “normal burst” pattern.

By integrating the clustering results, the public opinion trend lines shown in Figure 5, and the dynamic features of information propagation, we classified S2 as extreme burst, E2/S1/C1/C2 as normal burst, and E1 as long-tail, as shown in Table 5. Specifically, S2, triggered by a strong exogenous shock related to food safety concerns, exhibits only one single peak; the four peaks of E2, S1, C1, and C2 are densely clustered with multiple extrema, whereas E1, driven by recurring trust disputes, remains active for over 1100 h and undergoes multiple recurrences after complete dissipation, displaying a characteristic long-tail signature. Consequently, we adopted a three-cluster risk classification of ‘extreme burst, normal burst, and long-tail’ and propose the corresponding governance strategies.

#### 4.4.2. Robustness Testing and Validation

To assess the sensitivity of the K-means to random initial centroids and data noise, we conducted random-restart experiments and data noise experiments in the standardized feature space with k=3. Specifically in random-restart experiments, we varied the random seed across 100 trials, performing a single initialization per trial. Across these runs, the algorithm achieved an average silhouette coefficient of 0.344 (SD = 0.06), which exceeds the 0.30 threshold commonly taken to indicate meaningful—albeit still moderate—cluster separation and, therefore, confirms stable cluster geometry [52]. The mean adjusted rand index (ARI) was 0.95 (SD = 0.15), placing it in the “excellent agreement” category defined for ARI ≥ 0.90 [60,61].

Next, we performed noise-perturbation experiments by adding Gaussian noise (mean = 0, standard deviation = 0.05) to the original features, which achieved an ARI of 1.00 (SD = 0.00), demonstrating that the three-cluster structure remained completely unchanged under slight data fluctuations. Overall, K-means exhibited high robustness to initialization and minor noise, further supporting the reliability of the extreme burst, normal burst, and long-tail patterns.

### 4.5. Ablation Study of Propagation Patterns Under External Interventions

#### 4.5.1. Design and Results of the Ablation Study

When tracking the impact of major events on public opinion, information dissemination, and behaviors, ablation studies conducted by selectively including or excluding specific hotspot events and subsequently observing changes in the model’s goodness-of-fit can effectively identify events that play critical roles. Moreover, these ablation experiments allow for the quantification of the individual contributions of external interventions to the model’s predictive performance [62]. In practice, all potential stimulus points are initially labeled, after which the dataset is partitioned into subsets based on "inclusion versus exclusion" criteria. Each subset is then trained and evaluated using consistent model structures, hyperparameters, and training procedures. By comparing changes in goodness-of-fit across various scenarios, it becomes possible to pinpoint events significantly driving information dissemination pathways or shaping public behaviors. This approach not only reduces confounding biases arising from multiple simultaneous interventions but also enhances the robustness and interpretability of the conclusions.

As shown in Table 6, this study identifies two key intervention nodes from several representative public opinion events that significantly impact public attitudes, information dissemination, and behaviors. The original time series data are annotated accordingly, enabling the construction of four control subsets: Full Data, excluding First Intervention, excluding Second Intervention, and excluding All Interventions. The model uses retweets, comments, and likes as input features, with the public opinion intensity value as the prediction target, which is first normalized using MinMaxScaler. Subsequently, the normalized data are modeled using a three-layer LSTM network (containing 128, 64, and 32 units, respectively) followed by a two-layer fully connected network. The Adam optimizer is employed, with mean squared error (MSE) as the loss function. After inverse normalization of the predicted results, the $R^{2}$ goodness-of-fit is calculated under each scenario. The results indicate that the $R^{2}$ values for all events under the Full Data scenario are no lower than 0.80, demonstrating an excellent model fit and confirming the usability and effectiveness of the constructed model [63,64]. Furthermore, by comparing the changes in $R^{2}$ across different ablation scenarios, the study provides an in-depth analysis of both positive and negative impacts of key interventions on the model’s capability to accurately represent public opinion trends (see Table 7).

#### 4.5.2. Feature Analysis of Propagation Patterns Based on Ablation Results

The extreme burst pattern is typically triggered by strong exogenous shock. Using Event S2 as an illustrative example, multiple tanker trucks transported industrial oil to the edible oil market without proper cleaning, creating a serious food safety risk. This incident caused a rapid rise and subsequent swift decline in public opinion, characterized by a sharp, single-peak curve. In the ablation experiments conducted in this study, the model achieved a fit of $R^{2}$ = 0.86 under the Full Data scenario. After excluding the First Intervention, the $R^{2}$ value slightly increased to 0.91; however, after excluding the Second Intervention, it sharply decreased to 0.56. Even when both authoritative notifications were simultaneously excluded, the $R^{2}$ rebounded only to 0.70. These results indicate that the inter-ministerial joint investigation notification was the sole primary driver for the peak of public opinion in this incident, while other interventions made minimal contributions to the overall trend. Furthermore, the survey results from 666 participants demonstrate that authoritative, timely, and comprehensive information significantly reduces public risk perception and panic [65]. Therefore, in managing an extreme burst of public opinion, platforms should monitor peaks of public sentiment in real time. Competent authorities need to promptly issue comprehensive inspection reports and emergency response plans in a decisive manner early in an outbreak, distributing them on multilingual platforms such as Weibo, Douyin, and TikTok, accompanied by short videos or illustrative information, thereby anchoring public opinion and rapidly alleviating public panic.

The normal burst pattern typically shows jagged fluctuations, characterized by alternating local peaks, troughs, and sub-peaks during the outbreak period. Generally, public interest declines sharply after the initial exposure but then resurges following new rounds of topics or interpretations, resulting in two to three notable peaks and troughs. For example, Event E2 reached the hot-search list twice due to regulatory disclosures followed by an in-depth expose by Caixin. Event S1 triggered its first peak from the “315” exposure report on March 20 and subsequently regained attention due to expert commentary on March 23. Event C1 initially gained significant attention through an in-depth interview and was subsequently maintained by brand co-branding activities and fan interactions. Event C2 experienced multiple surges in public opinion driven by account blocking/unblocking and featured commentary. According to the ablation experiments, the Full Data scenario for Event E2 achieved $R^{2}=0.87$, rising slightly to 0.92 after excluding the First Intervention, and decreasing to 0.88 after excluding the Second Intervention. For Event S1, the $R^{2}$ under the Full Data scenario was 0.92, which decreased to 0.82 under excluding the Second Intervention. Event C1 exhibited an $R^{2}$ of 0.85 under the Full Data scenario but sharply fell to 0.23 after excluding the First Intervention. Event C2 showed an $R^{2}$ of 0.8262 under the Full Data scenario, which significantly decreased to 0.7609 after excluding the featured commentary, whereas excluding account blocking/unblocking slightly increased $R^{2}$ to 0.8457. These results distinctly highlight the differential impacts between “initial exposure” and “subsequent interpretation” in a normal burst of public opinion. To address this pattern, platforms should promptly release clarifying information, reinforced by expert interpretations and opinion leader dissemination, to reduce public hostility, enhance policy compliance [66], and significantly lower risk perception [67]. Additionally, it is recommended to set negative sentiment thresholds (e.g., a 20% negative emotion rate on Weibo) within monitoring systems, triggering immediate targeted interventions when exceeded, thus promptly stabilizing public sentiment.

The long-tail burst pattern can still be reactivated many times after the first peak is zeroed out. Taking Event E1 as an example, the public opinion intensity along its long-tail curve exhibited four distinct peaks. The first peak was triggered by the controversy surrounding the use of pseudo-site crab-catching videos in Northeast China. The second peak arose due to consumer disputes over cassava vermicelli being falsely marketed as sweet potato vermicelli. A delayed apology statement three days later triggered the third peak, and the final significant surge occurred when the regulatory bureau imposed a fine of 67.176 million yuan on the involved enterprise and ordered a production suspension, thus forming a typical “peak-zero-repeak” pattern. According to the ablation experiment results, the model achieved a high goodness-of-fit ($R^{2}$ = 0.87) under the complete dataset, indicating its strong ability to capture the overall trend. After excluding the First Intervention, $R^{2}$ decreased sharply to 0.71, demonstrating that the initial authoritative notification significantly influenced the sustained fermentation of public opinion. Conversely, excluding the Second Intervention (the apology statement) caused $R^{2}$ to slightly rise to 0.89, suggesting that while the apology statement briefly attracted attention, it mainly introduced noise and short-term fluctuations. Even when both interventions were excluded simultaneously, $R^{2}$ remained around 0.88, further confirming that the second statement was not the primary driver of the long-tail trend. Overall, the long-tail decay of this event predominantly relied on the initial authoritative information dissemination, while subsequent apologies only generated transient disturbances, insufficient to sustain continuous fluctuations. From the perspective of power-law decay, the persistent decline and repeated activation of public opinion depended more on spontaneous social-network diffusion and multiple external triggers rather than a single official response. Consequently, platforms should regularly release authoritative information on relevant topics to alleviate public anxiety [68]. Government agencies, media, platforms, and communities should collaborate proactively to swiftly address and clarify negative public opinion in the medium term, effectively shortening its persistence [69]. From a technical standpoint, platforms should prioritize the latest verified facts in real-time search results and provide detailed explanations via official channels such as Zhihu and Weibo Chat, thereby preventing secondary risks resulting from information vacuums.

## 5. Conclusions

This study develops a novel framework to quantify and analyze the “heat” of online public opinion by integrating user interactive behavior and emotional conflict. Through the application of information entropy and the information gain ratio, distinct weights are assigned to reposts, comments, and likes to capture their relative contributions to attention. An emotional variance metric is introduced to reflect the intensity of emotional conflict among netizens discussing the same event. We then combine these two metrics into a comprehensive heat index, which is decomposed using the HP filter to separate long-term trends and short-term fluctuations, extract six key quantitative features (number of peaks, time of first peak, maximum amplitude, decay duration, peak emotional conflict, and overall duration), and apply K-means to classify events into distinct dissemination patterns, which provides a data-driven basis for differentiating event-level behaviors. Finally, this study conducted ablation experiments on critical external intervention nodes to quantify their respective impacts on the propagation trend and to inform governance strategies. An empirical analysis of six representative public opinion events in 2024 demonstrates that this approach effectively reveals multi-round dissemination dynamics and the critical role of emotional drivers, thereby addressing limitations of traditional one-dimensional measures.

These findings reveal a substantial variation in dissemination patterns. Some events exhibit a rapid surge and a swift decline in attention (explosiveness). Others show multiple rebounds or an extended fermentation phases (cyclical evolution). As discussions deepen and emotions intensify, secondary or multiple waves of heightened attention may follow. Moreover, greater emotional conflict as measured by emotional variance strongly correlates with higher user interaction frequency and more sustained public interest, thereby increasing overall opinion heat. By combining interaction heat with emotional heat, this study employed feature extraction, clustering, and an ablation study to analyze the characteristics of different propagation patterns, and the framework provides fresh insights into how online public opinion forms and evolves. This method complements traditional approaches that rely only on interaction counts or sentiment polarity. These findings can help regulators and enterprises respond more effectively to unexpected opinion incidents, design targeted interventions, and improve management strategies.

However, this study relies primarily on textual data from the Weibo platform and does not incorporate other social media channels or multimodal information (e.g., images and short videos), limiting a comprehensive assessment of external influences and diverse sources of information. Additionally, the analysis focuses on internal social media dynamics and omits the quantitative modeling of external factors such as media coverage and official announcements. Sentiment analysis is based on SnowNLP and does not fully capture temporal emotional evolution. Future work will integrate multi-source and multimodal data, explore advanced dimensionality reduction or clustering techniques, and adopt sequence models to better capture dynamic emotional trajectories and multi-round dissemination patterns.

## Figures and Tables

**Table 1:** Public opinion event cases.

Category	Event ID	Event	Date
Enterprise Survival	E1	“Dong Bei Yu Jie” Livestream Sales Failure	4 September 2024–24 October 2024
	E2	“Crazy Xiao Yang” Livestreaming Meicheng Mooncakes	14 September 2024–29 September 2024
Social Livelihood	S1	315 Sausage Scandal	15 March 2024–25 March 2024
	S2	Oil Tanker Mixing Edible Oil Incident	2 July 2024–21 August 2024
Culture and Sports	C1	Li Ziqi’s Comeback	12 November 2024–27 November 2024
	C2	Wu Liufang Incident	22 November 2024–8 December 2024

**Table 2:** Key quantitative features and definitions.

Quantitative Feature	Definition and Explanation
Number of Peaks $\left(N_{p}\right)$	The total count of major peaks appearing in the comprehensive intensity curve (red line), used to determine whether the event undergoes multiple outbreaks.
Time of First Peak $\left(T_{p}\right)$	The hour at which the first major peak occurs, indicating how quickly the event reaches its initial peak within the dissemination timeline.
Maximum Amplitude $\left(A_{m}\right)$	The highest observed intensity value throughout the entire dissemination process, reflecting the strongest level of public attention or discussion.
Decay Time $\left(T_{d}\right)$	The time interval from the first peak to the first trough, illustrating how quickly the trend transitions from an increasing phase to a decreasing phase.
Peak Emotional Conflict $\left(C_{m}\right)$	The maximum variance in the emotional conflict index, capturing the greatest level of divergence or polarization in user sentiments during the event.
Overall Duration (D)	The total time span from when intensity first rises notably above the baseline until it returns to baseline, reflecting how long the event remains in public view.

**Table 3:** Key quantitative features of representative events.

Event ID	$N_{p}$	$T_{p}$	$A_{m}\;\left(\times 10^{-2}\right)$	$T_{d}\;\left(h\right)$	$C_{m}\;\left(\times 10^{-2}\right)$	D (h)
E1	13	28	0.551	432	4.975	1103
E2	8	29	3.047	16	3.567	290
S1	4	54	2.0348	53	1.843	117
S2	2	173	7.761	151	7.877	214
C1	5	31	6.864	28	5.711	183
C2	7	28	4.789	15	3.273	263

**Table 4:** K-means and Ward metrics.

*k*	K-Means		Ward	
	Inertia	Silhouette	Silhouette	CH
2	22.27	0.20	0.39	4.10
3	5.27	0.36	0.36	8.74
4	2.10	0.17	0.18	10.74
5	0.29	0.18	0.18	10.74

**Table 5:** K-means and Ward results.

Event	K-Means	Ward	Patterns
E1	C	1	Long-tail
E2	B	3	Normal burst
S1	B	3	Normal burst
S2	A	2	Extreme burst
C1	B	3	Normal burst
C2	B	3	Normal burst

**Table 6:** Time and description of external intervention in public opinion events.

Event	First Intervention		Second Intervention	
	Date	Description	Date	Description
E1	24 September 2024	Market Supervision Bureau of Benxi Manchu Autonomous County informs that the sweet potato vermicelli of “Dong Bei Yu Jie” has “no detectable sweet potato ingredients and detectable cassava ingredients”, and the other indexes are in line with food safety standards.	30 September 2024	“Dong Bei Yu Jie” issued an apology statement, saying that it has been sent to the national standard quality inspection department testing, and promised to all users a full refund.
E2	17 September 2024	Hefei High-tech Zone Market Supervision Bureau informed that “Crazy Xiao Yang” is suspected of misleading consumers with goods behavior and has filed for investigation.	19 September 2024	Caixin.com disclosed that “Crazy Xiao Yang” invited Hong Kong star Eric Tsang during a live broadcast and used “Hong Kong big brand” and “Michelin master modulation” to mislead consumers.
S1	20 March 2024	The Public Opinion Monitoring System (POMS) released a 315 exposure list and heat analysis, pointing out that “sausage” has become the hottest exposed food, driving a subsequent trend of public opinion.	23 March 2024	Titanium Media and other platforms published expert scientific articles to calm the controversy from the perspective of industry standards and nutritional value, effectively eliminating some misunderstandings.
S2	6 July 2024	China Agri-Industries Group issued a letter in response to “the quality of the brand involved is qualified” and cooperated with the investigation, trying to stabilize market confidence.	9 July 2024	Food Safety Office of the State Council and other seven ministries and commissions held a special meeting, set up a joint investigation team to investigate the whole chain, and informed that no other similar problems were found; the enterprises and personnel involved were to be punished.
C1	13 November 2024	CBNData interview: Li Ziqi revealed that the comeback is a temporary decision; in the past three years, she visited more than 100 intangible-heritage inheritors and will focus on cultural innovation in the future.	16 November 2024	On the first anniversary of the Zhejiang Rui’an Dongyuan Wooden Character Printing Cultural Research Institute and the launch of the Cultural IP Strategic Alliance, Li Ziqi was appointed “Cultural Communication Ambassador” and appeared at the ceremony.
C2	24 November 2024	After being banned for violating Weibo rules, the account was unbanned hours later, gaining over 1.2 million followers the same day, raising questions about the platform’s enforcement standards.	27 November 2024	The United Daily News published a commentary pointing out that the incident reflected the conflict between retired athletes’ career development and societal expectations, triggering a broader social discussion.

**Table 7:** Results of Ablation Study.

Event	Full Data	Excluding First Intervention	Excluding Second Intervention	Excluding All Interventions
E1	0.87	0.71	0.89	0.88
E2	0.87	0.92	0.88	0.89
S1	0.92	0.92	0.82	0.93
S2	0.86	0.91	0.56	0.70
C1	0.85	0.23	0.81	0.25
C2	0.83	0.85	0.76	0.79

Note. All values represent the coefficient of determination ($R^{2}$).

## Data Availability

The data presented in this study are available on request from the corresponding author.
